# Identifying archetypal cannabis consumers to inform drug policy design: a Q-sort assessment of young adults’ attitudes in Mexico City’s metropolitan area

**DOI:** 10.1186/s42238-021-00107-8

**Published:** 2022-01-08

**Authors:** Salvador Espinosa, Charles Marks, Gustavo Fondevila

**Affiliations:** 1 School of Public Affairs, San Diego State University, San Diego, USA; 2 School of Social Work, San Diego State University, San Diego, USA; 3 Department of Medicine, University of California, San Diego, San Diego, USA; 4 Department of Community Health Sciences, University of Reno, Nevada, Reno USA; 5 CIDE-Mexico, Mexico City, Mexico

**Keywords:** Cannabis, Motivations for use, Perceptions, Young adults, Q methodology, Mexico

## Abstract

**Background:**

As the legalization of cannabis moves forward in many countries, it is important to highlight the potential harm that excessive use can cause on young consumers. Crafting effective policy interventions to reduce the harm stemming from excessive use requires an understanding of the attitudes and motivations of young consumers.

**Methods:**

This article uses Q methodology to study four aspects of cannabis use among young adults from Mexico City’s metropolitan area: motivations for use, perceived consequences of use, reasons that would increase willingness to reduce consumption, and attitudes towards government regulation. A total of 110 cannabis users between 18 and 21 years old were recruited using chain-referral sampling. Using a Q methodology, we captured the relative importance that participants assigned to a series of statements and identified archetypal profiles of young adults who use cannabis for each of the four aspects mentioned above.

**Results:**

The sample for this research study included 76 men and 34 women. The average age of participants was 20 years old, and the average age when cannabis consumption started was 15 years old. For each of the four Q-sort factor analyses, we identified 4 distinct factors based on explained variance and interpretability. The Q factor analysis indicated that attenuation of a negative affect (i.e., anxiety, stress) and relaxation were primary motivations for cannabis use. Understood consequences of cannabis use ranged across aspect-archetype, reflecting legal (i.e., interacting with law enforcement), financial, familial (i.e., disappointing family members), and educational performance concerns. Participants indicated that finding alternative relaxation strategies, receiving credible evidence of the health harms of cannabis use, increased financial burden of purchasing, and increased inaccessibility of cannabis products would motivate reductions in use. Across archetypes, participants indicated a willingness to comply with cannabis policies which are simple and easy to understand, which do not lead to discrimination or law enforcement involvement, and which provide for legal places to purchase and use safe (i.e., free of adulterants) cannabis products.

**Conclusions:**

We posit that these archetypes could be useful to inform cannabis policy design. As the study reveals, participants’ cannabis use was primarily motivated by perceived improvements to mental health. Furthermore, participant responses indicated that they viewed cannabis use as a health matter, not a criminal one. Policies which aim to promote alternative mental health wellness and relaxation mechanisms, which aim to improve communication of potential health harms of cannabis, and which allow for the safe and legal purchase and use of cannabis may be effective in reducing cannabis-associated harms. Though our findings shed light on important aspects of cannabis users’ attitudes and perspectives, the sample size does not allow for a generalization of the findings and the drawing of conclusions about the population under scrutiny. Further research should consider the application of the Q methodology used in this article to a larger and more representative sample of cannabis users.

**Supplementary Information:**

The online version contains supplementary material available at 10.1186/s42238-021-00107-8.

## Background

Cannabis is one of the most consumed drugs across the globe. As of 2018, 192 million people worldwide (i.e., 3.9% of individuals aged 15–64) consumed cannabis, with use being substantially higher in North America than in other regions of the world (United Nations Office on Drug and Crime 2020). The prevalence of consumption is highest among young adults, such as in the USA where it is highest among young adults between 18 and 25 years of age (Center for Behavioral Health Statistics and Quality 2015). In 2019, it was estimated that 7.4% of youth aged 12–17 and 23% of young adults aged 18–25 in the USA had used cannabis in the prior month (SAMHSA 2019). In Mexico, the age at which consumption begins has gone from 20.6 years in 2002 to 17.8 years in 2016 (Instituto Nacional de Psiquiatría “Ramón de la Fuente Muñiz”, Instituto Nacional de Salud Pública, Comisión Nacional Contra las Adicciones, and Secretaría de Salud 2017). Furthermore, in Mexico, cannabis is the most consumed substance among individuals between 12 and 17 years old (Instituto Nacional de Psiquiatría, Comisión Nacional contra las Adicciones 2015).

While the failures of “War on Drugs” policies have prompted policy efforts to decriminalize the use of cannabis products, important concerns around the health impact of cannabis use on the developing brain remain. Frequent use during adolescence and early adulthood can lead to severe and persistent negative outcomes, such as problems with neurocognitive performance and alternations in brain functioning (Jacobus and Tapert 2014; Lubman et al. 2015; Hurd et al. 2020). Research in the USA also suggests that cannabis use may exacerbate depressive symptoms among youth experiencing depression (Weinberger et al. 2020; Gunn et al. 2020; Degenhardt et al. 2003). As this evidence shows, cannabis use may compound health harms experienced by a vulnerable population of adolescents and young adults. Given the prevalence of adolescent and young adult cannabis use and the growing availability of cannabis products in legalized and regulated markets, there is still a clear need for innovative and effective policy interventions to try to discourage excessive cannabis use among potentially vulnerable segments of the population.

We posit that policy interventions, which have generally focused on criminalization, interdiction, and law enforcement, have been ineffective because, to a great extent, they have failed to account for the motivations driving young adults to consume cannabis. Interestingly, recent research has indicated that individuals familiar with the most knowledge and information about cannabis tend to have more liberal attitudes about its use and, additionally, are more likely to consume it (Zeiger et al. 2020). There seems to be a dissonance between how cannabis use is conceptualized by policymakers and enacted into law versus how people who use cannabis view and understand their own cannabis use. While research indicates that perceived harmfulness has decreased significantly among adolescents (Keyes et al. 2016), it remains important to ensure that potential and current cannabis consumers are knowledgeable of the actual health consequences of excessive cannabis use. Taken together, there is a need for cannabis policy that accounts for the knowledge and attitudes of cannabis consumers and, further, which relies on an accurate understanding of the potential harms associated with excessive cannabis use.

The purpose of this study is to better understand the attitudes of young people who use cannabis in a country (Mexico) where discussions on legalization and regulation are taking place. We pose the following research questions: (1) what motivates young people to consume cannabis?; (2) what are their perceptions about the consequences of using it?; (3) under what circumstances would they be willing to reduce consumption?; (4) what would increase their willingness to comply with or adhere to programs aimed at reducing consumption? We apply Q-sort methodology to capture factors of greatest relative importance to each of these questions. We then apply Q-sort factor analyses to identify archetypal profiles of people who use cannabis (i.e., average cannabis consumers). Designing policy interventions to address cannabis consumption among the youth and young adults calls for a deeper understanding of their perceptions, attitudes, and motivations towards cannabis use and regulation. The findings of this study can provide improved insight into young adult cannabis consumers’ attitudes towards use and regulations, which may improve cannabis regulation efficacy. This research study contributes to a growing literature focusing on behavior-informed approaches to policy design (OECD 2017; Shafir 2013b; World Bank 2015), which draws insights from behavioral and social sciences to try to generate more effective policy interventions (World Bank 2015).

## Methods

This research study seeks to contribute to debates on drug policy design by using a Q-sort methodology (McKeown and Thomas 2013; Watts and Stenner 2005). The method allows the researcher “to discern people’s perceptions of their world from the vantage point of self-reference...[it] constitutes a methodology for the study of human subjectivity” (McKeown and Thomas 2013). As such, “[it] focuses on the subjective or first-person viewpoints of its participants” (Watts and Stenner 2005). Variations of the Q-sort methodology have been used in empirical studies on police perceptions (Chanin and Espinosa 2015), cannabis consumers’ attitudes regarding rule compliance (Espinosa 2019), studies on drug use prevention (Huang et al. 2020), and secondhand smoke exposure (Huang 2019).

While typical survey questionnaires ask participants to respond to distinct and independent questions, Q-sort asks participants to reflect on the relative importance of a series of factors in relation to a given prompt. For example, instead of asking participants how important various factors (i.e., relaxation, peer pressure, etc.) are in driving their cannabis use, the Q-sort approach is to ask participants which factors are most important, relative to one another. As such, Q-sort allows researchers to identify factors of greatest and least importance to a participant.

We posit that this methodology shall be seen as a complement to (and not a substitute of) studies relying on population-based samples, as it allows researchers to structure the viewpoints of cannabis consumers and build a cohesive narrative about their attitudes and motivations.

### Sample and data collection procedures

The Institutional Review Board at San Diego State University approved the research protocol for this research study. The protocol included specific measures to ensure the ethical treatment of human subjects, informed consent, and proper protection of the information.

Participants were recruited in the metropolitan area of Mexico City between April and October 2019 using chain-referral sampling. This is a non-probability method commonly used in the sampling of hidden populations. As Heckathorn explains, standard probability sampling methods can be inapplicable or prohibitively costly when the aim is to study hard-to-reach populations. This may occur because research subjects may be difficult to identify and recruit, have privacy concerns, or constitute a small part of the general population (Heckathorn 1997, 2002). Chain-referral sampling is often suitable when members of the target population know one another and are densely interconnected (Erickson 1979).

Initially, participants were identified by contacting people that the research team met during the exploratory stage of the project (Espinosa 2019) and during the initial phase of fieldwork for this particular research study. Information about the study was also disseminated through flyers that were placed where potential participants could see them (e.g., public places and offices where services to people who use drugs were offered). Recruitment efforts also included a chain-referral strategy by which study participants were asked to distribute the flyers among individuals who may be willing to join the research study.

All meetings took place in mutually agreed locations where safety and anonymity of the researchers and participants could be ensured. Potential participants were pre-screened to ensure that they qualified for the study and to receive detailed information about their role in the research study. Individuals qualifying for the study were reminded about the objectives of the study and asked to sign an IRB-approved informed consent form. Each participant received the equivalent of US$20 as compensation for their participation.

Fieldwork was divided into two phases: (1) the application of the Q questionnaire and (2) the semi-structured interviews (the questions/topics for the interview were defined based on the participants’ responses in the Q-sort exercise). This research study focuses on the results of the former.

### Experimental design

The Q-sort approach for this research study was designed by creating sets of cards containing statements related to each of the four research questions guiding this study (i.e., motivations for use, perceived consequences of use, reasons that would motivate consumers to reduce consumption, and attitudes towards compliance, and perceptions about complying)^1^^,^^2^. Each of the four decks of cards was placed in a separate envelope.

**Fig. 1:**
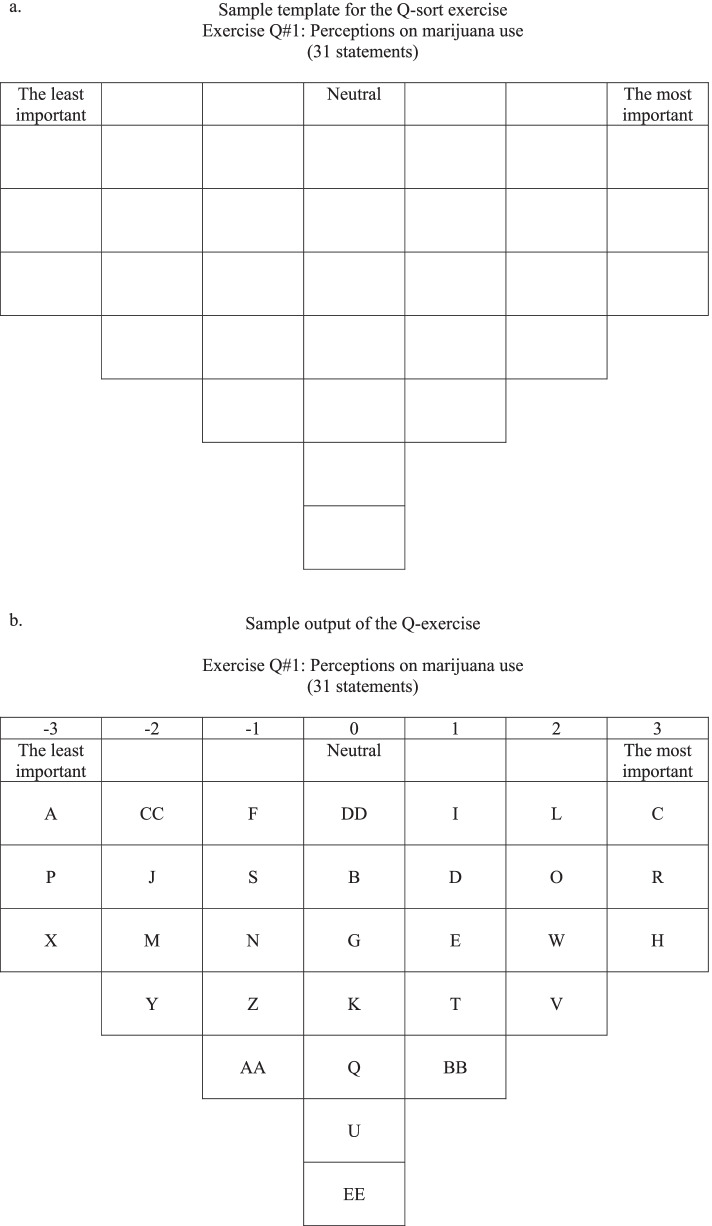
**a**, **b** Q-sort dynamic at a glance. **a** Sample template for the Q-sort exercise. **b** Sample output of the Q-exercise. Source: Espinosa (2019)

After responding to survey items capturing demographic information, each participant was then provided the first deck of cards (reflecting on motivations for cannabis use) and a corresponding Q-sort board (see Fig. 1a). Participants were provided the prompt “I use cannabis because...” and then instructed to organize the cards using the Q-sort board. Cards placed in the left-most column of the board correspond to the “least important” factors and cards placed in the right-most column correspond to “most important” factors. Each card had a distinct letter on the back. Once the participant completed the board, the researcher flipped the cards over (as seen in Fig. 1b) and recorded participant responses. This procedure was repeated for boards 2 (“What are the consequences of cannabis use?”), 3 (“What would cause you to reduce your cannabis use?”), and 4 (“I, as a cannabis consumer, would be willing to abide by the rules if...”).

The research team coded each set of responses by assigning a number to each column in the template. The statements located in the central column would be associated with a zero, those to the right to a positive integer (+1, +2, +3), and those to the left with a negative integer (−1, −2, −3). As such, for each board, participant responses were coded into a vector of the form [*a*,*b*,*c*,...] = [*+3*, −*2*, *0*, ...] where the letters represent each distinct card, and the numeric values represent where on the board the cards were placed. These vectors represent the unit of analysis for the Q-sort factor analysis we then employed.

For each of the four Q-sort boards, Q factor analysis was applied to identify archetypal response profiles. The Q factor analysis is a distinct methodology from what is typically referred to as “factor analysis” (McKeown and Thomas 2013). When a Q-sort board is filled out, the responses are relative to one another, and, thus, instead of treating each individual item on the board as a variable for analysis, we consider the relative arrangement of items (i.e., the participant’s completed Q-sort) as the unit of analysis. Q factor analysis is run, then, to reduce the sample of Q-sorts down to a predefined number of archetypal boards—the Q factor analysis identifies Q-sorts which are highly correlated with one another and then, by taking the average value of each item within the correlated Q-sorts, an archetypal response pattern (or a factor) is then generated. This archetypal response pattern represents the synthesis of highly correlated boards and can be understood to represent an archetypal person. The meaning extracted from each archetype is interpreted in a qualitative manner.

Specifically, given a set of *n* Q-sorts with *m* items (*q1*, *q2*, *q3*...*qm*) in them, Q factor analysis is executed as follows (McKeown and Thomas 2013). First, the number of factors to identify, *k*, is determined. Then, each board is represented as a vector where the value for each item is placed in the respective order (i.e., [1, −1, 0, 2...−2]). A correlation matrix is generated by calculating the inter-correlation between each Q-sort vector. Then, traditional factor analysis is applied to this correlation matrix to identify *k* factors. We applied a *varimax* rotation, commonly applied in Q-sort factor analysis, to identify distinct and differentiable aspect-archetypes (Watts and Stenner 2012). By identifying orthogonal (uncorrelated) factors with maximum variance, we were best able to capture such distinct aspect-archetypes. We further discuss this choice in the *Limitations*. For each factor, Q-sorts with a factor loading of at least 0.80 are considered to “load onto” that factor. For each factor, an archetypal Q-sort is generated by (1) taking each Q-sort that “loads onto” that factor, (2) calculating the average item score among each Q-sort for each of the *m* items, (3) sorting the items by the average score from lowest to highest, and (4) “filling out” the archetypal Q-sort from left to right (i.e., the item with the lowest score will be placed in the left-most column, the item with the median score will be placed in the middle column, and the item with the highest score will be placed in the right-most column) (McKeown and Thomas 2013). The end result is a set of *k* archetypal Q-sorts. The number of factors that load onto each board as well as the Eigenvalue and explained variance for each factor (from running the traditional factor analysis step) are extracted. The final number of *k* archetypal Q-sorts for each board was determined by the explained variance and qualitative interpretability. The Q factor analysis was executed using the “*qmethod*” package in the statistical software “R” (Zabala 2014).

### Participants

One hundred and ten cannabis consumers were recruited for this research study. As shown in Table 1, the average age of respondents was 20 years old and the average age when consumption started was 15 years old.^3^ Interestingly, 66% of participants mentioned that they were considering reducing their use, though we note that this value may be inflated because of a social desirability bias.

**Table 1:** Demographic and cannabis use characteristics of study participants (*n* = 110)

Age (in years)	
Average	20
Standard deviation	1.2
Gender	
Male	76 (69%)
Female	34 (31%)
Age at first use	
Average	15
Median	15
Standard deviation	1.9
Main motivation for first use (as % of total respondents)	
Curiosity	50.9
Friends	34.5
Others	14.6
Considering reducing consumption (as % of total respondents)	66
Main motivation for cannabis use	
Recreational	44
Medical	13
Addicted	48%
Sporadic user	3%

Source: Own calculations from participants’ self-reported data

## Results

### Analysis

Q factor analysis was used to extract information from the coded data. Tables 2, 3, 4, and 5, which summarize the findings of the quantitative analysis, illustrate the factors associated with each Q-sort and present their factor loadings, Eigenvalues, and explained variance. To assist in qualitatively interpreting each archetypal Q-sort, we present the most important (i.e., for board 1: +3 and +2) and least important (i.e., for board 1: −3 and −2) for each factor. For improved interpretability, we opted to define 4 archetypes for each Q-sort board (labeled A, B, C, D) and, across each board, these 4 archetypes explained approximately half the variation in participant responses.

**Table 2:** Results of the Q factor analysis: motivation for cannabis use

Archetype	Most important factors (factors: +2, +3)	Least important factors (factors: −2, −3)	Q-sorts loading	Eigenvalue	Exp. Var. (%)
A	● (+3) I like how it feels when I use it, it helps me relax and I feel creative and have interesting thoughts. ● (+2) It give me pleasure, it helps me feel good, and it helps me reduce stress, and sleep.	● (−3) Helps me forget my problems, I have a medical problem and helps me cope with depression. ● (−2) My friends also use it; it helps me feel I belong to the group I interact with; I stop thinking about my problems.	31	17.3	15.7
B	● (+3) It helps me relax, reduce stress, and sleep. ● (+2) It helps me feel less nervous, stop thinking about my problems and cope with depression & anxiety.	● (−3) My friends also use it; it helps me feel I belong to the group I interact with; I feel more attractive. ● (−2) I wanted to try something new; I feel braver and can have fun with friends who also use it.	24	14.9	13.5
C	● (+3) It helps me forget my problems, feel good, and reduce stress ● (+2) It is less harmful than other drugs, does not affect my health, helps me relax, feel less angry.	● (−3) I have a medical problem; I feel more attractive and braver ● (−2) It helps me feel less lonely, more confident and cope with depression and anxiety.	21	11.9	10.8
D	● (+3) It helps me forget or stop thinking about my problems and gives me pleasure. ● (+2) I like how it feels, it helps me feel less lonely, helps me relax and feel more spontaneous	● (−3) It is less harmful than other drugs; I have a medical problem; it helps me belong to the group I interact with. ● (−2) I wanted to try something new; my friends also use it; it does not affect my health and can fun with friends who also use it	10	7.8	7.1

Source: Own calculations from original data collected during fieldwork

**Table 3:** Results of the Q factor analysis: perceived consequences of cannabis use

Archetype	Most important factors (factors: +1, +2)	Least important factors (factors: −1, −2)	Q-sorts loading	Eigenvalue	Exp. Var. (%)
A	● (+2) I feel hungrier, have problems with the police, and spend more money than planned ● (+1) I am more introverted, have troubles remembering things, use more marijuana than planned, disappoint me loved ones, and go to work or school under the effects of marijuana	● (−2) I feel sick, have respiratory problems, and have problems with my significant other ● (−1) I don’t finish activities I start, do things I later regret, feel bad about myself, have troublesome thoughts and unwanted or risky sexual activities	28	14.0	12.7
B	● (+2) I feel hungrier, don’t finish activities that I start, and spend more money than planned ● (+1) I am more introverted, have troubles remembering things, feel tired or without motivation, and have troublesome thoughts	● (−2) I don’t show up at school or work, have problems with the police and with family members and friends. ● (−1) I don’t like talking to people, feel sick, don’t do well at school or at my job, drive a car under the effects of marijuana, and have unwanted or risky sexual activities	21	11.3	10.3
C	● (+2) I have troubles remembering things; have problems with family members and friends. My loved ones are disappointed. ● (+1) I don’t finish activities I start, use more marijuana than planned, and do things that I later regret, have troublesome thoughts. I feel bad about myself.	● (−2) I am more introverted, feel sick and can’t sleep. ● (−1) I don’t like talking to people, feel tired or without motivation, go to school or work, and drive a car under the effects of marijuana. Having unwanted or risky sexual activities.	14	10.3	9.3
D	● (+2) Driving while intoxicated, having problems with my significant other. My loved ones are disappointed ● (+1) I feel hungrier, don’t do well at work or school, do things that later regret, have unwanted or risky sexual activities, and spend more money than planned.	● (−2) I don’t like talking to people, have troubles remembering things, feel tired or without motivation. ● (−1) I am more introverted, can’t sleep, don’t face my problems, feel bad about myself, and have troublesome thoughts.	11	8.5	7.8

Source: Own calculations from original data collected during fieldwork

**Table 4:** Results of the Q factor analysis: reasons that would motivate participants to consider a reduction in cannabis use

Archetype	Most important factors (factors: +2, +3)	Least important factors (factors: −2, −3)	Q-sorts loading	Eigenvalue	Exp. Var. (%)
A	● (+3) If it is displayed in a place where I cannot reach it or if I know it has dangerous or disgusting ingredients. ● (+2) If I know that there are health risks associated with consumption, have less money to spend, receive convincing evidence of the negative consequences associated with use, or is demonstrated that it reduces sexual appetite (libido).	● (−3) If a public figure that I admire/respect recommends reducing consumption, my social group no longer uses marijuana. ● (−2) If the package containing marijuana has phrases highlighting health risks, the place selling it requires age verification, if I find different ways to deal with my problems, have friends who don’t use it.	39	17.7	16.1
B	● (+3) If I find other ways to relax, or it is displayed in a place where I cannot reach it. ● (+2) If I know there are health risks associated with consumption, find different ways to deal with my problems, if they start drug testing at my work, or the effects are no longer satisfying	● (−3) If the price starts increasing. I don’t care about taxes; I would consume the same quantity. ● (−2) If the place selling it requires age verification. I don’t care about price; I’d consume the same amount. If I can’t get it in the street anymore or if my social group is no longer using it.	15	10.5	9.5
C	● (+3) If the price is too high or I find other ways to relax ● (+2) If taxes I must pay to get it are too high, its effects no longer satisfy me, I have less money to spend, or have friends who don’t use it.	● (−3) If I am presented with convincing evidence about its negative consequences or if I know that it contains dangerous or disgusting ingredients. ● (−2) If the place selling it requires age verification, I am well-informed about the risks associated with use, a public figure that I admire/respect recommends reducing consumption, or if my workplace starts testing to know if I use it	10	7.8	7.1
D	● (+3) If I cannot get it in the street anymore, or the maximum amount I can obtain legally is enough to keep me away from illegal markets. ● (+2) The package contains phrases highlighting health risks, I can obtain it legally in various presentations (e.g., food, drinks), if there is a medicine to reduce my urge (desire) for it, or I know that it has dangerous or disgusting ingredients.	● (−3) The seller displays it where I see it but can't reach it, or it is demonstrated that it reduces libido (sexual appetite). ● (−2) The place selling it requires age verification, if I have friends who don’t use it, if I am well informed about the risks associated with use, my social group is no longer using it.	11	6.5	5.9

Source: Own calculations from original data collected during fieldwork

**Table 5:** Results of the Q factor analysis: reasons that would increase participants’ willingness to abide by rules regarding cannabis use

Archetype	Most important factors (factors: +2, +3)	Least important factors (factors: −2, −3)	Q-sorts loading	Eigenvalue	Exp. Var. (%)
A	● (+3) Police does not extort, detains or arrest me because I am a consumer. ● (+2) There are safe places to purchase marijuana, I am informed about the places where I can consume it and obtain it legally, and the rules with which I must comply are fair.	● (−3) If my family does not treat me like a sick person and people accept me as a consumer. I am not going to comply with any rule. ● (−2) If rules are easy to understand, complying with the rules is easy. If I am not forced to have addiction treatment, or if I am not discriminated for being a consumer.	28	13.5	12.2
B	● (+3) Rules are easy to understand, police do not arrest me for being a consumer and the rules I must comply with are fair ● (+2) Complying with the rules is easy are penalties for non-compliance are reasonable, I am not discriminated for being a consumer, and my community tolerates responsible consumption.	● (−3) I am not going to comply with any rule. If I am not forced to have addiction treatment. If employers and government stop requiring drug tests. ● (−2) Regulation does not limit my freedom of choice or the varieties of marijuana that I can purchase. If I can legally purchase good-quality marijuana. If the government does not keep records showing that I am a consumer.	20	10.2	9.3
C	● (+3) There are safe places to purchase it, I am not discriminated for being a consumer, I can purchase good-quality marijuana legally. ● (+2) There are safe places to consume, authorities do not treat me like a criminal, police do not extort me for being a consumer, and people accepts me as a consumer	● (−3) Marijuana that I get legally is cheaper than what I currently get. If employers and government stop requiring drug tests. I am not complying with any rule. ● (−2) Rules are easy to understand, penalties for non-compliance are reasonable. I can obtain it via social clubs or similar organizations. The government does not keep records showing that I am a consumer.	16	9	8.2
D	● (+3) Rules are easy to understand, I can consume it privately, and purchase good-quality marijuana legally. ● (+2) There are safe places to consume it, complying with the rules is easy, my community tolerates responsible consumption, I am informed about the places where I can consume it	● (−3) My family does not treat me like a sick person. If I am not detained by police. I am not going to comply with any rule. ● (−2) If authorities don’t treat me like a criminal, police don’t extort me for being a consumer, if I am not discriminated for being a consumer, there are no limits to the quantity I can legally purchase.	9	7.5	6.9

Source: Own calculations from original data collected during fieldwork

### Q1: Motivations for cannabis use

The first Q-sort asked participants to reflect on factors that motivated them to use cannabis (see Table 2). Across all four identified archetypes, relaxation was identified as a top reason for using cannabis, though there are important variations between archetypes. Archetype A, which 31 participants loaded onto, listed liking how they feel, relaxation, stress reduction, creativity, and sleep improvement as their top motivations for cannabis use. Archetype B, which 24 participants loaded onto, listed promoting relaxation; reducing stress, depression, and anxiety; aiding with sleep; and problem avoidance as their top motivations for cannabis use. Archetype C, which 21 participants loaded onto, listed feeling good, promoting relaxation and sleep, reducing stress and feelings of anger, and feeling cannabis is less harmful than other drugs as their top motivations. Archetype D, which 10 participants loaded onto, listed problem avoidance, liking how it makes them feel, feeling more spontaneous, and relaxing. These top motivations appear to fit into two categories: enhancing positive mental states (i.e., enjoying how it feels to use cannabis) and mitigating negative mental states (i.e., self-treating stress and anxiety). For Archetypes A, C, and D, we observe top motivations including both, and for Archetype B, we observe top motivations pertaining entirely to mitigating negative mental states. Of importance, this indicates that participants from all archetypes report self-treating stress, anxiety, depression, and sleep deprivation—this indicates that policy efforts aimed at reducing adolescent and young adult cannabis use should aim to provide alternative options for individuals to seek improved mental health. Interestingly, every archetype listed social reasons (i.e., using cannabis to fit in with friends, because friends use it, to feel less lonely) in their set of least important reasons for their cannabis use.

### Q2: Perceived consequences of using cannabis

The second Q-sort asked participants to reflect on the consequences of using cannabis (see Table 3). Some of the results reported are common symptoms associated with cannabis consumption (e.g., feeling hungry, having troubles remembering things). Others, however, unveil information about consumers that policymakers may not always take into consideration. Archetype A, which 28 participants loaded onto, listed problems with the police, spending too much money, becoming introverted, disappointing loved ones, and attending work/school intoxicated as important consequences. Archetype B, which 21 participants loaded onto, listed spending too much money, inability to finish activities, becoming introverted, feeling tired, and having troublesome thoughts as primary consequences. Archetype C, which 14 participants loaded onto, listed having problems with family and loved ones, not being able to finish activities, and feeling bad about themselves as important consequences. Archetype D, which 11 participants loaded onto, listed driving while intoxicated, problems with family and significant other, struggling with school, doing things they later regret, and taking risky sexual behaviors as important consequences of cannabis use. Interestingly, when taken as a whole, these results unveil that young cannabis consumers are aware of some of its physiological effects, but also about the ways in which it affects their social connections, and personal finances (e.g., spending more money than planned). We may understand, though, that cannabis consumers are willing to continue the use of cannabis *despite* these consequences—which provides further indication that cannabis consumers’ motivations for using cannabis (*Q1*) may outweigh the perceived consequences of use.

### Q3: What would motivate reduction in cannabis use?

The third Q-sort asked participants to reflect on what would motivate them to reduce cannabis use (see Table 4). Archetype A, which 39 participants loaded onto, listed cannabis being displayed out of reach, knowing if it has been laced with dangerous ingredients, knowing there are health risks associated with use, receiving *convincing* evidence of the negative consequences of use, and if it reduces libido as top motivators for reduction. Archetype B, which 15 participants loaded onto, listed finding different ways to relax or deal with problems, cannabis being displayed out of reach, knowing there are health risks, cannabis no longer being satisfying, and drug testing at work as primary motivations. Archetype C, which 10 participants loaded onto, listed the price being too high or not having enough money, finding other ways to relax, cannabis no longer being satisfying, or having friends who do not use cannabis as primary motivators. Archetype D, which 11 participants loaded onto, listed not being able to get it on the street anymore, being able to legally buy the amount they want, packaging which highlights the health risks, if there is medication to reduce the desire for cannabis, and knowing if cannabis is laced with anything dangerous as primary motivators. These factors can be related to three necessary (though not sufficient) conditions to be pondered during policy design: accessibility, information, and personal finances. We may understand these conditions as leverage points to promote cannabis use cessation—noting, though, based on our earlier findings (*Q1*) that individuals reported using cannabis to mitigate negative health effects (i.e., reducing stress and anxiety, promoting sleep). This is important because a policy initiative aimed solely at reducing cannabis consumption without addressing motivations for cannabis use may fail to have the intended consequences (for example, increasing cannabis taxes without providing alternative stress relief options may drive some people who use cannabis to purchase it from illegal markets).

The least important reasons noted across archetypes for this Q-sort also contain important policy-related information. Common strategies used in tobacco control, such as age verification, ID requirement, and placing products out of reach were consistently identified as of least importance across archetypes. Interestingly, Archetype B reported price being of the least importance. In contrast, Archetype C listed price as the most important, instead identifying potential health consequences as least important. This indicates that a one-size-fits-all approach to cannabis regulation will likely be ineffective at promoting reductions in cannabis use.

### Q4: Factors that would promote willingness to comply with cannabis regulations

The fourth (and final) Q-sort addresses the issue of compliance directly: what situations would increase their willingness to abide by rules regarding cannabis use (see Table 5). Across all four identified archetypes, there were key similarities as participants generally reported they would be willing to comply with cannabis regulations if those regulations were easy to understand and fair, if they were not discriminated by their community and law enforcement for their cannabis use, and if they know where they could safely purchase and use cannabis. Archetype A, which 28 participants loaded onto, listed not being extorted, arrested, or detained by police for being a consumer; having a safe place to purchase cannabis; and the rules being fair as motivators to comply. Archetype B, which 20 participants loaded onto listed the rules being easy to understand and fair, punishment for non-compliance being reasonable, not being arrested for being a consumer, and not being discriminated (including by own community) for consumption as motivators to comply. Archetype C, which 16 participants loaded onto, listed having a safe place to purchase and consume cannabis, being able to purchase high-quality cannabis, not being treated like a criminal or extorted by police, and not being discriminated against as top motivators to comply. Archetype D, which 9 participants loaded onto, listed the rules being easy to understand and comply with, being able to consume cannabis in private or in safe places, and their community being tolerant of their use as top motivators to comply. Participants generally indicate that their use should not be subject to criminal justice intervention—taken together with participant’s desire for cannabis regulations to be “fair,” we can postulate that most participants will view any law enforcement involvement in cannabis regulation as “unfair.” This is consistent with prior results (*Q1*) which indicate that participants report various health benefits as primary reasons for cannabis use—individuals who conceptualize their cannabis use as, at least in part, medicinal may view law enforcement involvement in cannabis compliance as inappropriate. While ensuring compliance (especially in situations where, like in some US states, purchase and use are now legal) is a complex issue, keeping the consumers’ perspective in mind could contribute to design more balanced policies and strategies where rights, responsibilities, and sanctions are clear and straightforward. It is valuable to note that across all archetypes, the “I am not complying with any rule” factor was identified as of least importance. This indicates that in the presence of fair regulations, participants across all archetypes are willing to comply with those regulations.

## Discussion

This research study was aimed at identifying archetypal profiles of young adults consuming cannabis in Mexico City’s metropolitan area. The research team gathered information about participants’ motivations for using cannabis, their perceived consequences of use, factors that would motivate them to reduce consumption, and their attitudes towards cannabis regulations. The findings of this research study further our understanding of the motivations driving this segment of the population to initiate cannabis use (Fales, Ladd & Magnan 2019; Lee et al. 2009; Dumbill et al. 2020). The results of the Q methodology used in this article enabled us to start crafting narratives reflecting the consumer’s vantage point, not only in terms of their motivations for using cannabis, but also for other relevant aspects that influence their decisions to consume it.

Having a good understanding of the attitudes and motivations of different types of cannabis consumers (in the case of this study, young adults) is necessary to improve the effectiveness of regulations and drug policies. This is especially important given that regulatory agencies are in a unique position to alter cannabis consumption patterns and mitigate potential harms to public health.

As the results of the Q factor analysis show: first, that study participants were primarily motivated to use cannabis to improve physical and emotional well-being, either through the enhancement of positive affect or the mitigation of negative affect; second, that all participants were conscious of many of the consequences of cannabis use, such as negative effects on social relationships and the financial cost, and, further, it is implied that they continue cannabis use despite these consequences; third, participants indicated a range of factors that would motivate a reduction in use including (but not limited to) the accessibility of cannabis and the availability of convincing evidence of the harms of cannabis use; and, finally, participants articulated that the rules being “fair,” not being stigmatized for their cannabis use, and not being subject to law enforcement as primary reasons they would comply with cannabis regulations. Taken together, results indicate that many young cannabis consumers in our sample view their cannabis use as having a tangible benefit in their life (*Q1*) despite the understood negative consequences associated with consumption (*Q2*). Their willingness to comply with cannabis regulations appears to be dependent on their perceptions about the “fairness” of those regulations and not being targeted by law enforcement (*Q4*). Many participants reported that if presented with “convincing” evidence of the health harms of cannabis would encourage them to try to reduce cannabis use (*Q3*). These findings offer useful insights that policymakers should take into consideration when developing cannabis reduction interventions.

While there has been evidence in the USA that portraying cannabis as a medical product has resulted in more permissive attitudes towards cannabis use, this is likely due to the evolving understanding of cannabis and the resultant shifting public attitudes. For decades, cannabis has been depicted as a dangerous and illicit substance of abuse—the modern depiction of cannabis as a medicinal product likely indicates to people that the use of cannabis is less dangerous and harmful than once thought. It is logical then that the decriminalization and medicalization of cannabis would result in more permissive attitudes. This should not inherently be considered a negative outcome, as public attitudes of cannabis (shaped by the drug war) have been shaped by the politics of criminalization, as opposed to facts about cannabis itself. As public attitude naturally shifts towards being more permissive of cannabis use, it will be important to provide accurate information (as opposed to Reefer Madness fearmongering) about the potential harms of cannabis use to help individuals avoid excessive use.

Furthermore, given that participants are reporting that they use cannabis to alleviate negative affect, it is reasonable to hypothesize that policies which aim to limit access to cannabis without promoting either (1) access to medicinal cannabis or (2) access to alternative coping strategies of equal or greater effectiveness may be viewed as threats to their immediate well-being. As such, cannabis policies which focus on reducing potentially harmful use while permitting medicinal use will likely be viewed favorably by cannabis consumers. If the reduction of cannabis use is a policy goal, then it is important that such policies make alternative coping strategies available. Furthermore, research should be undertaken to determine if proposed alternatives are sufficient to promote reductions in cannabis use. It is also possible that cannabis use is the best available treatment for noted symptoms and policymakers should be transparent if they are unable to provide an alternative. It may be beneficial then to focus on upstream interventions which aim to alleviate sources of stress and depression given that these appear to be primary drivers of cannabis use.

Also, the narrative stemming from this Q-sort research study sheds lights on perceptions that policymakers often ignore or bypass. For example, for young consumers participating in this research study, the advice of a public figure they admire or packages highlighting health risks are not perceived as important factors to encourage a change in their consumption (Table 4). Given that participants indicated that learning about the harms of cannabis use may motivate reductions in their use, it is important to identify the best and most trustworthy avenues through which to disseminate information to cannabis consumers. As research has indicated that cannabis consumers generally have greater knowledge about cannabis use than those who do not use it (Zeiger et al. 2020), messaging that comes from people who use cannabis may be viewed as more credible by other cannabis consumers.

The Q-sort method is subject to the limitations of a typical self-report study, though is designed to reduce the potential impact of social desirability, strategic behavior, or other self-serving biases. Despite changing perceptions about cannabis, it remains an illicit product in many countries, raising questions about the reliability of information that may be subject to social desirability or self-serving biases. Q-sort methodology reduces the probability of strategic behavior, as it presents all possible responses to a particular question simultaneously and relative to one another. By requiring participants to rank statements relative to one another, they are able to address the research prompt without needing to directly answer questions about potentially stigmatizing behaviors, attitudes, or beliefs.

Our study has additional limitations that we should note. First, while respondent-driven sampling is understood to be an effective strategy for recruiting hard-to-reach populations, the ability to fully capture the population of interest is dependent on the interconnectedness of the population. As such, our approach likely missed individuals with the fewest social connections (i.e., no one to refer them into the study)—as such, future research efforts focusing on cannabis use should attempt to recruit individuals with minimal or no social networks, as these individuals may be particularly susceptible to the harms of cannabis use and may hold differing attitudes towards use. While the sample for this research study was sufficient to draw inferences from the Q factor analysis, further research could replicate the methodology by using a larger and more representative sample of young consumers. This is particularly important for harder-to-reach populations, such as homeless youth, who may be subject to increased exposure to health harms and legal consequences of use and, as such, may have distinct attitudes and perceptions around cannabis use. Additionally, research should aim to expand upon and refine the items used to populate each Q-sort board to ensure that this approach captures all relevant latent factors influencing participant responses. Also, we chose the *varimax* rotation to extract distinct and relevant (i.e., maximizing number of load Q-sorts) archetypes to improve result interpretability. Alternative approaches include using the (non-orthogonal) centroid method or other orthogonal rotations such as *quartimax*. While the centroid method can (correctly) capture the correlation between factors, orthogonal rotations generally result in a “simple structure” that is more readily interpretable (McKeown and Thomas 2013). Furthermore, we note that even though varimax factors are orthogonal, each participant Q-sort is correlated (i.e., non-zero) with each factor. As such, it is important to articulate that our identified archetypes are intended to capture distinct participant profiles which can be readily interpreted to inform policy decisions, not to suggest that participants loading onto a factor are inherently unrelated to those who load onto another factor (i.e., these factors represent informative cut-points along a spectrum of responses). Also, whereas the *varimax* rotation is ideal for identifying distinct factors, the *quartimax* rotation is better suited for a “general factor” which explains a majority of variance (i.e., a majority of Q-sorts load onto this “general factor”) (Akhtar-Danesh 2017). Given our objective to identify distinct and relevant profiles, we determined that the *varimax* rotation was preferential to other methods.

## Conclusion

This research study used a Q-sort method to gather information about the perceptions of 110 young cannabis consumers from Mexico City’s metropolitan area. The findings reported in this article provide insight into the reasons why they choose to consume it, the perceived consequences of using it, the factors that they would motivate them to reduce consumption, and factors that would increase their willingness to comply with rules and regulations pertaining to cannabis use. This research study sheds light on aspects of consumers’ belief system that may end up affecting the effectiveness of policy interventions aimed at reducing cannabis use among the youth. Our findings indicate that cannabis consumers are motivated to use cannabis to alleviate negative affect (i.e., depression, stress) and that they may be willing to reduce cannabis consumption provided alternative coping strategies and/or convincing evidence of the harms of cannabis use. Educational initiatives seeking to promote the harms of cannabis use should address the harms of competing harms such as stress and depression. Furthermore, participants consistently reported willingness to abide by cannabis regulations if they are “fair” and not treated as a criminal matter.

## Supplementary Information

**Additional file 1.** A copy of the form used during data collection.

## Data Availability

De-identified data generated or analyzed during this study, as well as the R code used for the statistical analysis, are available from the corresponding author on reasonable request.

## Footnotes

1 A copy of the form used during data collection that includes the statements included in each card is provided in the Supplementary information.

2 To be able to define the information to be included in the Q-sort exercise, the principal investigator used the self-assessment tool “E-Marijuana Check Up to Go” as reference point (see https://sacd.sdsu.edu/cps/resources/checkup-marijuana. Last retrieved on March 1, 2021) and conducted extensive meetings with users and addiction specialists.

3 While statistics about cannabis use in Mexico indicate that consumption among teenagers has been steadily growing over time, human subject protection protocols made it difficult to consider individuals under 18 years old in this study.

## References

[CR1] Akhtar-Danesh N. A comparison between major factor extraction and factor rotation techniques in Q-methodology. Open J Appl Sci. 2017;7(4). 10.4236/ojapps.2017.74013.

[CR2] Center for Behavioral Health Statistics and Quality (2015). National survey on drug use and health 2014.

[CR3] Chanin J, Espinosa S. Examining the determinants of police department transparency: the view of police executives. Crim Justice Policy Rev. 2015:1–22. 10.1177/0887403415596039.

[CR4] Degenhardt L, Hall W, Lynskey M (2003). Exploring the association between cannabis use and depression. Addiction.

[CR5] Dumbill EW, Hanewinkel R, Degge H, Ezekwe E, Nnajiofor M. Cannabis use motivations: a study of young adults in Nigeria. Drugs Educ Prev Policy. 2020. 10.1080/09687637.2020.1834514.

[CR6] Erickson B (1979). Some problems of inference from chain data. Soc Methodol.

[CR7] Espinosa S, Siddiki S, Espinosa S, Heikkila T (2019). What comes after marijuana legalization? An exploratory assessment of users’ attitudes towards rule compliance. Contextualizing compliance in the public sector: individual motivations, social processes, and institutional design.

[CR8] Fales JL, Ladd BO, Magnan RE. Pain Relief as a Motivation for Cannabis Use Among Young Adult Users With and Without Chronic Pain. The Journal of Pain 2019;20(8):908–16. 10.1016/j.jpain.2019.02.001. 10.1016/j.jpain.2019.02.001 30735731

[CR9] Gunn RL, Stevens AK, Micalizzi L, Jackson KM, Borsari B, Metrik J (2020). Longitudinal associations between negative urgency, symptoms of depression, cannabis and alcohol use in veterans. Exp Clin Psychopharmacol.

[CR10] Heckathorn DD (1997). Respondent-driven sampling: approach to the study of hidden populations. Soc Probl.

[CR11] Heckathorn DD (2002). Respondent-driven sampling II: deriving valid estimates from chain-referral samples in hidden populations. Soc Probl.

[CR12] Huang, Chiu-Mieh, et.al. (2019). Patterns of parents’ perspectives on protecting young children from secondhand smoke exposure: a Q-methodology study. J Adv Nurs Pp. 2591-2602. 10.1111/jan.14029 10.1111/jan.14029 30993733

[CR13] Huang C-M (2020). Perspectives emerged from students and supervisory staff interaction in drug use prevention: a Q methodology investigation. Int J Environ Res Public Health.

[CR14] Hurd, Y., et al. (2020). Cannabis and the developing brain: insights into its long-lasting effects. J Neurosci, 39(42). https://doi.org/10.1523/JNEUROSCI.1165-19.2019 10.1523/JNEUROSCI.1165-19.2019 PMC6794936 31619494

[CR15] Instituto Nacional de Psiquiatría “Ramón de la Fuente Muñiz”, Instituto Nacional de Salud Pública, Comisión Nacional contra las Adicciones, & Secretaría de Salud. Encuesta Nacional de Consumo de Drogas, Alcohol y Tabaco 2016-2017: Reporte de Drogas. Mexico, DF; 2017. 10.21149/17295 41150976

[CR16] Instituto Nacional de Psiquiatría/ Comisión Nacional contra las Adicciones. Encuesta Nacional de Consumo de Drogas en Estudiantes 2014: Reporte de Drogas. Mexico, DF; 2015.

[CR17] Jacobus J, Tapert S (2014). Effects of cannabis on the adolescent brain. Curr Pharm Des.

[CR18] Keyes KM (2016). How does marijuana policy affect US youth? Medical marijuana laws, marijuana use and perceived harmfulness: 1991-2014. Addiction.

[CR19] Lee CM, Neighbors C, Hendershot CS, Grossbard JR. Development and preliminary validation of a comprehensive marijuana motives questionnaire. J Stud Alcohol Drugs. 2009;70(2):279–87. 10.15288/jsad.2009.70.279. 10.15288/jsad.2009.70.279 PMC2653613 19261240

[CR20] Lubman DI, Cheetham A, Yücel M (2015). Cannabis and adolescent brain development. Pharmacol Ther.

[CR21] McKeown B, Thomas DB (2013). Q methodology.

[CR22] OECD. Behavioral insights and public policy: lessons from around the world. Paris; 2017.

[CR23] SAHMSA (2019). National Survey of Drug Use and Health (NSDUH). Substance abuse and mental health services administration.

[CR24] Shafir E (2013). The behavioral foundations of public policy.

[CR25] United Nations Office on Drug and Crime. World drug report. Vienna; 2020.

[CR26] Watts S, Stenner P (2005). Doing Q methodology: theory, method and interpretation. Qual Res Psychol.

[CR27] Watts S, Stenner P (2012). Understanding the analytic process: factor rotation and the preparation of factor arrays. Doing Q methodological research: theory, method and interpretation.

[CR28] Weinberger A, et al. Cannabis use among youth in the United States, 2004-2016: faster rate of increase among youth with depression. Drug Alcohol Depend. 2020;209(1) 10.1016/j.drugalcdep.2020.107894. 10.1016/j.drugalcdep.2020.107894 32126453

[CR29] World Bank. World development report 2015: mind, society, and behavior. Washington, DC; 2015.

[CR30] Zabala A (2014). qmethod: a package to explore human perspectives using Q methodology. R J.

[CR31] Zeiger J (2020). Attitudes towards cannabis mediate the relationship between cannabis knowledge and use in active adult athletes. J Cannabis Res..

