# Supplementary material for: Identifying archetypal cannabis consumers to inform drug policy design: a Q-sort assessment of young adults’ attitudes in Mexico City’s metropolitan area
Source: J Cannabis Res. 2022 Jan 8;4:5. doi: 10.1186/s42238-021-00107-8 (PMC8742343; doi:10.1186/s42238-021-00107-8)
Supplement: Supplementary file 1 — Additional file 1. A copy of the form used during data collection. [file 42238_2021_107_MOESM1_ESM.pdf]

SAN DIEGO STATE UNIVERSITY
School of Public Affairs

ID# ___________________

A Q-sorting Study on Rules Perceptions among Marijuana Users in Mexico and the United States

Instructions for the interviewer:

	Checked?
• **Don't forget to assign a code prior to the interview (ID#)**	
• Give participants a brief explanation of the study and the terms of his/her involvement.<br>• Ask if he/she has any questions.	
• Make sure the person is at least 18 years old (ask for proof of age).	
• Read "informed consent form".<br>• Determine if the person is cognitively able to participate in the study by applying a post-consent quiz (if not, thank him/her and end the interview)	
• If the interviewer determines that the person is able to participate, ask him/her to sign the consent form **or** to give his/her consent orally (in this case, tape-record the answer)	

Section 1 Semi-structured interview

1.1 Questions to estimate periodicity of use and socio-economic background

	**Interviewer's notes**
1. In what city do you live?	• City: __________________
2. How old are you?	• Age: _____
3. Since this study seeks to obtain information from current users of marijuana, I need to start with the following question: When is the last time you used marijuana or cannabis in any form?	• Last time consumed: __________<br>• Has user consumed it within the past 12 months? (Circle one: YES NO)
4. Approximately how many days per week do you use marijuana?	• Times per week: _____
5. Let us assume that "one unit" of marijuana is about the size of a cigarette. How many units of marijuana do you use every day?	• Units per day: ______
6. What is your current occupation?	• Current occupation: ______

1.2 Users' perceptions on use

	Interviewer's notes
7. When did you start using marijuana?	
8. Why did you start using marijuana?	
9. Have you ever considered reducing your use of marijuana? (In other words, use less than what you currently use)	
10. Can you think about reasons why you would consider reducing your use of marijuana?	
11. Which of the following statements best describes you?<br><br>a. I use marijuana for recreational purposes<br>b. I use marijuana for medical reasons<br>c. I am addicted to marijuana<br>d. I use it sporadically<br><br>What are the other reasons why you use it?	• Circle answer: (a) (b) (c) (d)<br><br>• Other reasons for use? _______________

Section 2 Q-sorting exercise

Q-soring EXAMPLE

This research study will allow us to learn about the views of users of marijuana about legalization, regulation, and other factors. To do so, I will ask you to rank several phrases in terms of their importance, compared to other phrases listed. The example below illustrates how the process works.

The box below lists nine words that are thought to explain an athlete's performance.

Table 1.

Height				
		Nutrition		Coordination
			Speed	
				Attitude
	Sleeping habits			
Strength				Team player
		Water consumption		

Lets assume that I ask you the following question: What are the factors that contribute to an athlete's success?

You will be asked to sort the words by using a chart that look like this:

Table 2.

Most important		Neutral		Least important

Please rank the words appearing in table 1 by writing them in table 2. You can only write one word in each space.

Do you have any questions?
Can we proceed?

SAN DIEGO STATE UNIVERSITY
School of Public Affairs

ID# ___________________

2.1 Q-sorting Q#1: Perceptions on marijuana use

I use marijuana because…

A. It helps me forget my problems
B. It gives me pleasure
C. It helps me feel good
D. It is less harmful than other drugs
E. It is easy to obtain
F. I have a medical problem
G. I wanted to try something new
H. I helps me feel less lonely
I. My friends also use it
J. I don't feel the same effect when using other drugs
K. I like how it feels when I use it
L. It does not affect my health
M. It helps me reduce stress
N. It helps me relax
O. It helps me feel more confident
P. I feel more confortable when I talk to people
Q. I feel less bored
R. It helps me feel that I belong to group with whom I interact
S. I can have fun and be with friends who use marijuana
T. It helps me cope with depression
U. It helps me cope with anxiety
V. It helps me feel less angry
W. I stop thinking about my problems
X. I feel creative and have interesting thoughts and ideas
Y. It helps me sleep
Z. I feel more attractive
AA. I feel more spontaneous
BB. It is just a habit that I have
CC. I feel more energetic
DD. I feel more brave
EE. It helps me feel less nervous

Q#1

I use marijuana because…

Least important reason			Neutral			Most important reason

ID# ___________________

2.2 Q-sorting Q#2: Perceptions on the negative side effects of consumption

Question: Which of the following do you experience as a result of your marijuana use?

A. I am more introverted
B. I don't like talking to people
C. I feel sick
D. I cough and have respiratory problems
E. I feel more hungry
F. I have troubles remembering things
G. I can't sleep
H. I feel tired or without motivation
I. I don't do well at school or at my job
J. I don't finish the activities that I start
K. I go to school or work under the effects of marijuana
L. I don't show up at school or work
M. Driving a car under the effects of marijuana
N. I have problems with the police
O. I don't face my problems
P. I use more marijuana than I had planned
Q. My loved ones are disappointed
R. Doing things I later regret
S. I feel bad about myself
T. I spend more money than I had planned
U. Having problems with my significant other
V. Having problems with my family members and friends
W. Having troublesome thoughts
X. Having unwanted or risky sexual activities (e.g. not using a condom)

<u>Q#2</u>

Perceived consequences of using marijuana…

The least important consequence		Neutral		The most important consequence

2.3 Q sorting #3: When would users consider reducing consumption?

Question: Under what circumstances would you consider reducing your use of marijuana?

A. If I discover health risks associated with marijuana use
B. If I see pictures showing the consequences of using marijuana
C. If the taxes I must pay when purchasing marijuana are too high
D. If the price of marijuana is too high
E. If the price of marijuana starts increasing
F. If the package containing the marijuana I purchase has phrases highlighting the health risks
G. If the place selling marijuana displays it in places where I can see it, but it is out of my reach
H. If the place selling marijuana asks for my age verification
I. If the effect of the marijuana is no longer satisfying
J. I don't care about the price of marijuana. I would consume the same quantity that I use right now
K. I don't care about taxes. I would consume the same quantity that I use right now
L. If I have less money to spend
M. If I find different ways to deal with my problems
N. If I find other ways to relax
O. If I have friends who don't use marijuana
P. If I am well informed about the risks associated with marijuana use (e.g. using marijuana and alcohol at the same time)
Q. If I am presented with convincing evidence of the negative consequences associated with marijuana use
R. If a public figure that I admire and/or respect recommends reducing consumption
S. If I cannot get it on the street anymore
T. If marijuana is more potent
U. If my social group is no longer using marijuana
V. If I can obtain it legally in various forms (e.g. food, drinks, etc.)
W. If I can access it legally with a medical prescription
X. If the maximum amount that I can obtain legally is enough to keep me away from illegal markets
Y. If there is a medicine that helps me reduce my urge (desire) to use marijuana
Z. If there is another product with similar effects than marijuana
AA. If it is demonstrated that it reduces sexual appetite (libido)
BB. If they start testing if I use drugs at my workplace
CC. If I know that the marijuana I use has dangerous ingredients (e.g. pesticides) or disgusting ingredients (e.g. fertilizer/manure)

Q#3

Circumstances under a participant would consider using less marijuana…

**The least important circumstance**			**Neutral**			**The most important circumstance**

2.4 Q-sorting D: Perceptions on regulation and compliance

Instructions: Lets assume that purchasing marijuana becomes legal, but the government requires users to comply with certain rules. Please organize the information based on the importance that you give to each phrase

Question: Under what circumstances would you be more willing to comply with rules pertaining to the purchase of marijuana?

A. Regulation does not limit my freedom of choice
B. Regulation does not limit the varieties of marijuana that I can purchase
C. There are places to safely consume marijuana
D. The marijuana that I get legally is cheaper than what I currently get
E. Rules are easy to understand
F. Complying with the rules is easy
G. I am not going to comply with any rule
H. Penalties for not complying with the rules are reasonable
I. Authorities do not treat me like a criminal
J. There are places to purchase marijuana safely
K. Strategies dealing with marijuana consumption are similar to the existing ones for alcohol or tobacco
L. My family does not treat me like a sick person
M. Police does not try to extort me because I am a consumer
N. Police do not arrest me for being a consumer
O. Regulation allows me to grow my own marijuana
P. I am not forced to have addiction treatment
Q. I can obtain marijuana via social clubs or similar organizations
R. I am not discriminated for being a marijuana consumer
S. My community tolerates responsible consumption
T. The law does not limit the quantity of marijuana that I can legally purchase
U. I am not detained by police
V. I am informed about the places where I can consume marijuana
W. I can obtain marijuana legally
X. I can keep consuming marijuana privately (e.g., in my own house)
Y. I can buy it legally at a good price
Z. People accept me as a marijuana consumer
AA. If employers and the government stop requiring drug tests
BB. The rules with which I must comply are fair
CC. I can legally purchase good-quality marijuana
DD. The government does not keep records showing that I am a marijuana user

Q#4

The participant would be more willing to comply with rules related to marijuana if…

**The least important reason**			**Neutral**			**The most important reason**

=== We have finished. Thanks so much for your participation===
